# Governance, financial development and China’s outward foreign direct investment

**DOI:** 10.1371/journal.pone.0270581

**Published:** 2022-06-30

**Authors:** Chen Gao, Ya Wen, Deyong Yang

**Affiliations:** 1 China Economics and Management Academy, Central University of Finance and Economics, Beijing, China; 2 School of Economics, Beijing Technology and Business University, Beijing, China; The Bucharest University of Economic Studies, ROMANIA

## Abstract

Deeply investigating the relationship between governance, financial development, and outward foreign direct investment (OFDI) is beneficial to formulating effective policies to accelerate Chinese firms’ pace of overseas expansion. Based on the theoretical mechanism analysis, this paper empirically analyzes the impact of Asian governance and financial development on China’s OFDI using the panel data of 37 Asian countries from 2003 to 2017. The results show that the host country governance has a negative and statistically significant impact on China’s direct investment in Asia. The conclusion remains valid even after overcoming the interference of endogenous and economic cycle fluctuations. Moreover, using the mediating effect model, we find that financial development is an important channel through which host country governance affects China’s OFDI. In further discussion, the findings suggest that with the scale of OFDI expanding, the role of governance takes an inverted "U" shape, and the "Belt and Road" initiative (BRI) weakens the negative impact of governance quality on China’s OFDI. Furthermore, governance has shown more remarkable restraint on China’s OFDI in neighboring, coastal, and low-income countries in the heterogeneity test. ​From the perspective of host country governance, this paper provides more specific guidance to formulate China’s direct investment policy in Asia.

## 1. Introduction

The “going out” strategy implemented by China in 2000 has brought tremendous development potential to enterprises’ outward foreign direct investment (OFDI) [1]. Specifically, China’s “Belt and Road” initiative (BRI) in 2013 has made the foreign investment activities of enterprises more vigorous [2]. Most countries in Asia have participated in the Belt and Road Initiative and are important markets for Chinese enterprises to invest abroad. These countries are not only China’s neighbors but also relatively similar in terms of language and culture. China has established good diplomatic relations with Asian countries [3].

In 2020, China’s investment in Asia was $112.34 billion, a year-on-year growth of 1.4%, accounting for 73.1% of China’s total OFDI (UNCTAD, 2021). Besides, Non-financial direct investment by Chinese companies also mainly flows to Asian countries, where more than half of the industrial parks along the “Belt and Road” are concentrated. However, in OFDI, firms will be affected by the host country governance [4]. There are numerous cases where poor governance reduces the investment confidence of Chinese overseas companies, e.g. SAIC Motor failed to acquire South Korea’s SSANGYONG Group in 2004, the US government blocked Huawei’s planned acquisition of 3Leaf in 2011, and Chinese companies’ investment in Southeast Asia suffered severe losses due to the unrest in Vietnam in 2014. These failed cases illustrate that host country governance quality has become a critical factor that cannot be ignored in corporate OFDI and directly influences the location choice and scale of investment [5].

This paper is motivated by the fact that the previous studies have focused on “the Belt and Road” countries and African countries. They did not consider the impact of Asian governance on foreign direct investment before, lacking evidence of theoretical models and analysis of transmission mechanisms. Therefore, this paper fills this gap by testing the internal relationship between host country governance, financial development and China’s direct investment in Asia. On the one hand, we try to construct a model considering multinationals and governance heterogeneity. On the other hand, we make an empirical test based on panel data on China’s direct investment in Asian countries from 2005 to 2019.

The main contributions are as follows: first, in terms of research perspective, this paper profoundly studies the impact of Asian governance on China’s OFDI, exploring the effective mechanism which enriches the relevant literature on foreign direct investment research. Second, as for research methods, we establish a theoretical model to analyze the nexus between host country governance and OFDI. On the basis of benchmark regression, the distance between the country and the equator is used as an instrumental variable to eliminate endogeneity, and financial development is introduced as an intermediary variable to explore the influence channels of governance on China’s OFDI. Third, we proceed with the quantile and exogenous tests to examine the heterogeneous influence of different investment scales and governance under the BRI. Fourth, regarding the heterogeneity analysis, Asian countries are divided into China’s neighbors and non-neighbors, landlocked and coastal, high-income and low-income groups, respectively. Thus to examine the impact of host country governance on China’s direct investment in different regions.

The rest of this paper is organized in the following way: The second section is literature review and theoretical analysis, and puts forward research hypotheses on this basis. The third section provides model specification and data and the fourth section analyzes the empirical results. The fifth section discusses the relationship between the quality of governance and OFDI according to the heterogeneity test. We put an end to this work through a conclusion.

## 2. Literature review and theoretical analysis

### 2.1. Literature review

Scholars have investigated the impact of host country governance on OFDI from various perspectives [6–8]. Many research demonstrated that high-quality governance encourages more foreign direct investment inflows [9, 10]. To be specific, the high-quality governance of host country represents a stable political environment and relatively complete legal institutions, providing an orderly and low-risk investment environment for foreign investors [11]. Indeed, the excellent governance environment of host country enables foreign-funded enterprises to obtain market information in a timely and efficient manner, thereby reducing the probability of losses and sunk costs caused by information asymmetry [12]. Shleifer and Vishny found that foreign corporations encounter higher political risks in host countries with poor governance, such as expropriation of assets, exchange restrictions, political disputes, and war risks [13]. Poor governance breeds corruption and rent-seeking problems. Therefore, only investors paying bribes to the government can obtain administrative approval permits, which will induce investors to increase operating costs and lower production efficiency [14]. Moreover, because of the hidden nature of corruption, it will bring more significant distortions than tax increases [15].

Nonetheless, the influence of host country governance on China’s OFDI is still controversial. Cheung et al. have investigated the determinants of China’s direct investment in Africa from 1991 to 2005. Their results showed that Chinese corporations are more inclined to invest in countries that are politically stable and highly democratic [16]. Chen et al. noted that natural resources are not the only determinants of China’s OFDI inflow in Africa, and host country governance also has a considerable positive impact on China’s investment [17]. Deng concluded that the legal institution stimulates China’s investment in developing countries but has no apparent effects in developed countries based on the spatial econometric model [18]. For the period 2002–2011, Wang et al. analyzed the 842 OFDI transactions of China, and reported that host country governance, government efficiency, regulatory quality, and corruption control are helpful to OFDI in China, while investors tend to avoid countries with stricter legal institutions [19]. He and Xu used the Heckman selection model and investment gravity model to detect the influencing factors of China’s investment in countries along the BRI from 2003 to 2015 [20]. They argued that a stable political situation and a high rule of law are attractive to foreign capital inflows.

In contrast, studies have also proved that the impact of governance quality on China’s OFDI is contrary to traditional theories [21–23]. Pan and Jin employed panel data set of 117 countries over the period 2003–2013 to detect the conduction mechanism and effect of bilateral political relations and governance on OFDI in China. They showed that Chinese outbound companies prefer countries with better political relations and poor governance, as good bilateral relations can mitigate the negative effects of political risks and the uncertainty brought about by a backward governance environment [24]. Meng and Dong have denoted that China-friendly countries’ political and social risks have no apparent inhibitory effect on China’s OFDI. However, companies will choose low-risk countries that are not friendly to China [25]. However, some scholars have revealed that host country governance has no significant impact on the location selection of OFDI [26, 27]. These contradictory findings may be explained because these researchers select different samples or periods, and governance characteristics show complexity and diversity [28].

The financial development of host country is an essential reference for OFDI location selection [29]. Financial developments can affect FDI flows in liquidity, financial execution of contracts, and transaction costs [30]. However, findings on the relationship between financial development and capital inflows are inconsistent. On the one hand, host countries’ financial development and service efficiency are positively correlated with the quantity and quality of FDI inflows [31]. Likewise, developed financial markets are beneficial to accelerating the conversion rate of savings to investment and strengthening risk management and technological innovation to improve the efficiency of capital accumulation and allocation in the financial system [32–34]. At the same time, financial development reduces financing costs and investment risks [35, 36], thereby increasing manufacturers’ profit expectations and triggering capital inflows [37].

On the other hand, Albuquerque developed a basic institutional framework to explore the relationship between financial development and FDI flows based on the incomplete institutional hypothesis, finding that financial development and FDI location choice are mutually alternative [38]. Economies with lower levels of financial development tend to rely more on FDI to make up for the host country’s insufficient funds [39, 40]. The massive inflow of FDI is a substitute for the host country’s lagging and distorted financial system, but this substitution effect will not last long [41]. Moreover, the development of financial credit markets will also dampen this substitution effect [42]. Finally, many scholars have scrutinized the relations between financial development and foreign direct investment from a nonlinear perspective [43, 44].

As regards the nexus between governance and financial development, sound governance is critical to fostering financial development as it establishes and maintains property rights, effective legal institutions and financial supervision [45, 46]. Many scholars have obtained sufficient evidence to support that the quality of governance has a meaningful impact on financial development [47–49], especially for low-income and state-fragmented economies [50]. Cai documented that the progress of financial development in different countries varies according to the quality of governance. Governance quality is positively correlated with financial development [51]. Indeed, good governance can optimize the efficiency of financial resource allocation and enable enterprises to acquire convenient financing channels. Chinn and Ito showed that financial systems benefit more from a higher-quality governance environment, but only after governance reaches a certain threshold level [52]. This situation is more prevalent in low-income economies, most of which are still on the side of lower governance quality, indicating that further improvements in governance quality can promote the high-quality development of financial markets [53].

### 2.2. Theoretical model

This paper constructs a model considering multinationals and governance heterogeneity, investigating the role of host country governance nested into institutional theory on China’s OFDI. It mainly includes the following three steps: first, based on maximizing consumers’ utility in the host country, we construct a constant elasticity of substitution utility function of residents in the host country. Second, according to the producer behavior theory, we establish the production function and calculate the optimal pricing of multinationals’ sales in the host country. Finally, from the perspective of multinationals’ OFDI decision-making, we employ the probability equation to judge the relationship between host country governance and OFDI when profits are maximized.

There is no monetary department in this model, and all income and expenditure are priced in kind. We assume that there is only one monopolistic competition in the host country’s market, and N companies in the industry produce differentiated products; that is, each company can only produce one specific product. This inquiry refers to the method of Melitz [54] to establish the model in which labor, capital, and intermediate product inputs are simultaneously used in the production process. There is a total of L consumers in the host country, and each can provide a unit of labor; the average wage is w; K indicates the total capital, and the interest rate is *r*. The total income of the host country is expressed as:

W=wL+rK (1)

#### 2.2.1. Consumer preference maximization

The consumer utility function is set in the form of a constant elasticity of substitution (CES) function:

$$U={\left(\int_{0}^{N}q{\left(i\right)}^{\frac{\sigma -1}{\sigma }}\mathrm{di}\right)}^{\frac{\sigma }{\sigma -1}}$$ (2)

where N refers to the consumption collection of heterogeneous products, q(i) represents the consumer’s consumption of the product, σ devotes the elasticity of substitution between differentiated products, and σ>1. Under the condition of budget constraints, consumers pursue the maximization of utility, which is expressed by the following combination function:

$$\begin{array}{c} \max U={\left[\int_{0}^{N}q{\left(i\right)}^{\frac{\sigma -1}{\sigma }}di\right]}^{\frac{\sigma }{\sigma -1}}\\ \;s.t.\;W=\int_{0}^{N}p(i)q(i)\;di \end{array}\}$$ (3)

where *p*(*i*) is the price of a single product *i*; *W* represents the income of the whole society. We can easily obtain the optimal consumption of the product as follows:

$$q(i)=\frac{p{\left(i\right)}^{-\sigma }}{P^{1-\sigma }}W$$ (4)

where $P=[{\int_{0}^{N}{p(i)}^{1-\sigma }di]}^{\frac{1}{1-\sigma }}$ is the overall price index.

#### 2.2.2. Producer profit maximization behavior

Referring to the findings of Law et al. [55], it is essential to explain financial development from the governance perspective. Governance is positively correlated with financial development [56]. Only when the quality of governance is relatively complete can credit activities be carried out smoothly, and the interests of shareholders can be guaranteed. The model assumes that governance devotes to reforming and deepening the financial system. So, the model assumes that the host country’s budget for financial development is $\bar{h}$, fixed during the inspection period. *α* represents host country governance, and the function of financial development is as follows:

$$t=t(\bar{h}+\alpha )$$ (5)

where *t*′>0 and *t*′′<0. It indicates that the improvement of governance ability can promote financial development. Moreover, the financial development of the host country is conducive to the production efficiency of the investing country. We suppose that the output of multinationals that make direct investment in the host country not only depends on their effective labor, capital, and intermediate inputs, but also on the level of financial development of the host country. The return to scale is unchanged during the production process, and the production function is set as follows:

$$y(i)=t\beta l{\left(i\right)}^{\iota }k{\left(i\right)}^{\kappa }m{\left(i\right)}^{1-\iota -\kappa }$$ (6)

where *l*(*i*)、*k*(*i*)、 *m*(*i*) respectively expressed as the number of labor, capital, and intermediate product inputs employed by multinationals. *ι* and *κ* represent the output elasticity of labor and capital, and 0<*ι*, *κ*<1. *β* is labor productivity, and obeys the distribution function *Z*(*β*) and *Z*′>0.

We change Eq (6) into the production function form of compound input:

y(i)=tβG(i) (7)

where $G(i)={l(i)}^{\iota }{k(i)}^{\kappa }{m(i)}^{1-\iota -\kappa }$ indicates compound input. Let *f* be the input price of the intermediate product, then the compound input cost is:

c(w,r,f)=min{wl(i)+rk(i)+fm(i)} (8)

The net profit of FDI by multinationals is:

π(i)=p(i)y(i)−c(w,r,f)G(i)−F (9)

where *F* is the fixed cost required for multinationals’ OFDI. When the economy reaches equilibrium, the demand and output are equal, that is, *q*(*i*) = *y*(*i*). Substituting Eqs (4), (6), (7), and (8) into the Eq (9), the optimal pricing of the enterprise that obtains the conditions for maximizing profit is:

$$p(i)=\frac{\bar{n}c(w,r,f)}{t\beta }$$ (10)

where $\bar{n}=\sigma /(\sigma -1)$ refers to the price markup in a monopolistic competitive market.

#### 2.2.3. Decisions on OFDI of multinationals

We take Eqs (1), (4), (5), (6), (7), (8), (10) into Eq (9), and then the equation is:

$$\pi (\beta )=\frac{1}{\sigma }{\left[\frac{\bar{n}c(w,r,f)}{t(\bar{h}+\alpha )\beta P}\right]}^{1-\sigma }W-F$$ (11)

For simplification, we assume that the host country’s market is independent of other countries’ markets. Pursuing high profits is the most fundamental driving force of multinational OFDI. Therefore, multinationals’ decision-making on OFDI relies on whether the net profit obtained in that country is *π*(*β*)>0. The equation can be adjusted to:

$$\beta >{\left(\frac{\sigma F}{W}\right)}^{\frac{1}{1-\sigma }}\frac{\bar{n}c(w,r,f)}{t(\bar{h}+\alpha )P}$$ (12)

Due to the above assumptions, firm productivity *β* obeys the distribution function *Z*(*β*), and we can get the probability equation of foreign direct investment of multinationals as follows:

$$\begin{array}{l} \mathrm{Pr}[\pi (\beta )>0]=\mathrm{Pr}[\beta >{\left(\frac{\sigma F}{wL}\right)}^{\frac{1}{1-\sigma }}\frac{\bar{n}w}{t(\bar{h}+\alpha )P}]\\ \;=1-Z[{\left(\frac{\sigma F}{wL}\right)}^{\frac{1}{1-\sigma }}\frac{\bar{n}w}{t(\bar{h}+\alpha )P}] \end{array}$$ (13)

Taking the partial derivative of governance level from Eq (13), we can get:

$$\frac{\partial \mathrm{Pr}[\pi (\beta )>0]}{\partial t}\frac{\partial t}{\partial \alpha }=z(\beta ){\left(\frac{\sigma F}{wL}\right)}^{\frac{1}{1-\sigma }}\frac{\bar{n}w}{P}t{\left(\bar{h}+\alpha \right)}^{-2}t^{\prime }(\bar{h}+\alpha )$$ (14)

From formula (14), we can find that the partial derivative is greater than 0. It means that the higher governance of the host country, the more likely it is to attract foreign investment.

Governance is a key determinant of a country’s financial and economic development [57]. Specifically, good governance promotes well-functioning financial systems ensuring funding to the most effective investments [58]. Meanwhile, sound financial development can reduce enterprises’ financing constraints, attracting foreign investment [29]. Therefore, combined with the derivation results of the above theoretical model, it can be seen that financial development is an important channel for host country governance to affect the FDI of the investing country’s enterprises.

### 2.3. The actual situation of China’s OFDI

We attempt to further analyze the mechanism of how the governance level of Asian countries affects China’s OFDI. The relevant theories on OFDI are based on the experience of investors in developed countries, hence there are inevitably differences in the analysis applied to emerging economies, and we should critically examine the applicability of general theories when studying OFDI. The governance structure of emerging economies determines the ability and willingness of multinational enterprises to invest overseas. The traditional FDI theory shows that a better governance environment in the host country provides enterprises with a more stable investment environment and sound property legal protection [59]. However, a large body of scholars has obtained conclusions contrary to traditional theories in the research on China’s OFDI [60, 61]. Zhang and Fei found that both China’s OFDI in developed and developing countries showed the characteristics of “institutional risk preference”, using panel data of 113 countries over the period 2003–2017 [62].

According to existing literature research, the reasons can be stated as follows: First, government-led SOEs do not prioritize profit maximization while making OFDI [21]. Second, most Asian countries are developing countries, where the level of government governance is relatively poor. At the same time, China is more likely to take advantage of complex social relationships to exert its comparative advantage in OFDI in such countries [63]. Third, China’s institutional system is not sound enough. Most multinational companies have also grown up in this environment, so they have strong adaptability to invest in economies with poor governance. Since multinational corporations in developed countries have already occupied large markets in regions with relatively high levels of governance, Chinese corporations choose nations with relatively low levels of governance to invest in to access as many overseas markets as possible [64]. Fourth, the imbalance between supply and demand in China’s labor market has led to rising domestic labor costs, an important factor driving multinational companies’ overseas investment. Given this, Chinese companies will shift investment to countries with low levels of governance to seek low-cost labor [61].

Meanwhile, governance capacity is generally considered to be one of the important factors affecting financial development, and the level of governance contributes to financial development [51, 65]. Additionally, the influence of finance on corporate OFDI has not reached a consistent conclusion. Most scholars believe that the financial development of the host country has a significant role in promoting corporate OFDI [66, 67], whereas Liu and Gao have shown that the effect of the host country’s financial development on corporate OFDI has the characteristics of regional and economic development heterogeneity [68]. The good financial development of the host country not only brings low financing costs and investment risk to Chinese multinational enterprises, but also has a market crowding effect. The entry of Chinese enterprises will create a competitive relationship with similar local enterprises, and will also increase the uncertainty of the success of corporate investment [69]. In addition, the increase in the number of local enterprises will also have a substitution effect on Chinese multinational enterprises.

In light of the literature review and modeling framework, China’s direct investment in Asian countries is incorporated in the theoretical model, and the following assumptions are proposed:

Hypothesis 1a: The level of governance in the host country positively affects China’s direct investment in Asia.

Hypothesis 2a. Financial development is an important channel through which the level of governance promotes China’s direct investment in Asia.

Based on the actual situation of China’s OFDI, this paper puts forward:

Hypothesis 1b: The level of governance in the host country has an inhibitory effect on China’s direct investment in Asia.

Hypothesis 2b: Financial development is an important channel through which the level of governance inhibits China’s direct investment in Asia.

## 3. Methodology

### 3.1. Model specification

The main objective of this paper is to analyze the role of governance in the location selection of OFDI for Chinese firms. This paper draws on the research method of Yuan et al. [70], then we take logarithmic form for all variables and construct a multivariable linear regression model. The regression equation will be as follows:

$${lnOFDI}_{it}=\gamma_{0}+\gamma_{1}{WGI}_{it}+\sum_{j}^{T}\gamma_{j=2}{control}_{it}^{j}+\gamma_{i}+\delta_{t}+\varepsilon_{it}$$ (15)

where lnOFDI_it_ is the stock of China’s OFDI in country *i* at time t. We use the stock of OFDI rather than the flows as the dependent variable, mainly for the following two reasons: on the one hand, the flows of China’s investment in Asian countries change greatly every year, and the data for many years is less than zero, which is not helpful to empirical analysis. On the other hand, there are many missing values of OFDI flows. Therefore, we choose China’s OFDI stock as the dependent variable to ensure the data’s continuity and integrity. In addition, we logarithmized the data for the dependent variables to remove heteroskedasticity.

WGI_it_ refers to the host country governance, which includes six indicators, namely political stability and absence of violence, rule of law, government effectiveness, regulatory quality, voice and accountability and control of corruption. *γ*_*i*_ and *δ*_*t*_ represent country fixed effects and time fixed effects, respectively. ε_it_ designates the model error term. $\sum_{j}^{T}\gamma_{j=2}{control}_{it}^{j}$ are the set of control variables:

lnGDP devotes to market scale that is approximated by log of real GDP. Seeking economies of scale and maximizing output benefits are the key reasons why China is investing abroad [21].

lnDIS represents bilateral distance cost. Using linear distance has the following defects: First, distance costs are alterable due to transportation costs. Second, when estimating the gravity model with fixed effect, the distance will be treated as an individual fixed effect and thus cannot be identified. To overcome these problems, we use the product of bilateral distance and international oil price to express the bilateral distance cost [6, 71], that is: *lnDIS* = d_ij_×P_oil price_, with $d_{ij}=(\sum_{k\in i}\left({pop}_{k}/{pop}_{i}\right)\sum_{l\in j}({pop}_{l}/{pop}_{j})d_{kl}^{\theta }{)}^{1/\theta }$ and i and j denote the host country and China, respectively. d_kl_ refers to the geographic distance between the major cities of the two countries, pop_i_, pop_j_ respectively represent the total population of the two countries, pop_k_, pop_l_ respectively denote the population of the main cities of the two countries, Θ represents the elasticity of bilateral distance. For the convenience of calculation, we set Θ = 1.

lnLAB indicates labor endowment measured by the labor force participation rate over 15 years old. Multinational corporations prefer host countries with low labor costs when choosing a location for outbound investment [72].

lnFDI denotes foreign investment openness approximated by log of net FDI inflow (% of GDP). Generally speaking, a host country with a high degree of openness to foreign capital is favorable for foreign-invested enterprises to carry out economic activities, for its domestic investment environment provides favorable conditions for foreign capital inflow [73].

lnINF refers to macroeconomic stability, measured by the inflation rate [28]. Inflation causes distortions in relative prices that prevent investors from making sound investment decisions. In addition, high inflation rates tend to check the export performance of domestic and foreign investors and thus inhibit export-oriented FDI by increasing the prices of locally sourced inputs [21].

lnRES represents the host country’s total rent of natural resources as a share of GDP [74]. Harnessing the natural resources of host countries is one of the drivers of China’s foreign direct investment. Referring to existing research, China has invested more money in oil- and mineral-rich Asian countries [16].

lnFAC indicates infrastructure that is approximated by the average of fixed telephone users (per 100 people), fixed broadband users (per 100 people), and mobile cellular users (per 100 people). The complete infrastructure can improve the production efficiency of enterprises by reducing the cost of information collection and transmission.

lnTRA refers to trade openness expressed by the sum of imports and exports which are relative to GDP. The host country of an open economy is easily known to investors, and trade barriers will increase the transaction cost of investors. Therefore, high trade openness will encourage companies to invest.

### 3.2. Data

Given the availability and integrality, the data in this article does not include Hong Kong, Macau and Taiwan, and finally chose panel data of China’s direct investment in 37 Asian countries that covers the period 2005–2019. The specific list of 37 countries is as follows: Uzbekistan, Yemen, Israel, Iraq, Iran, Qatar, India, Indonesia, Kyrgyzstan, Kazakhstan, Turkmenistan, Turkey, Tajikistan, Korea, Bangladesh, Nepal, Pakistan, Bahrain, Brunei, Sri Lanka, Singapore, Japan, Cambodia, Saudi Arabia, Thailand, Kuwait, Jordan, Myanmar, Laos, Philippines, Mongolia, Vietnam, Afghanistan, The United Arab Emirates, Oman, Malaysia and Lebanon.

In regard to data sources, the stock data of China’s direct investment in Asia came from the Statistical Bulletin of China’s Outward Foreign Direct Investment over the years; the geographic distance data between the major cities of the two countries was collected from the CEPII Database; international oil price data, foreign direct investment data, and economic development indicators were sourced from the International Monetary Fund (IMF); governance quality indicators were from Worldwide Governance Indicators (WGI), and the remaining data all came from the World Bank’s statistics. We rely on Table 1 to sum up the definitions of the variables and their source. Meanwhile, we show the stock of China’s direct investment in Asian countries from 2005 to 2019 in S1 Appendix. We can find that China’s direct investment stock in Asian countries shows an upward trend, but there is obvious heterogeneity in regional distribution. China’s investment stock in the ASEAN region accounts for a relatively high proportion. In 2019, China’s stock of direct investment in Singapore accounted for the highest proportion, followed by Indonesia, Laos, and Malaysia. The countries with less investment stock in China include Lebanon, Bahrain, Oman, etc., which shows that it is necessary for us to conduct a heterogeneity analysis.

**Table 1:** Data description and source.

Variables	Description	Source
lnOFDI	China’s OFDI stock in Asian country i in year t	Statistical Bulletin of China’s Outward Foreign Direct Investment
WGI	The variables of governance quality	WGI
CC	Control of corruption	WGI
GE	Government effectiveness	WGI
PS	Political stability and absence of violence	WGI
RQ	Regulatory quality	WGI
RL	Rule of law	WGI
VA	Voice and accountability	WGI
lnGDP	Host country market size measured by log of real GDP	WDI
lnDIS	Bilateral distance cost measured by log of the product of the bilateral trade distance and the average international oil price	CEPII, IMF
lnLAB	Labor endowment measured by the labor force participation rate over 15 years old	WDI
lnFDI	Foreign investment openness measured by log of net FDI inflow (% of GDP)	IMF
lnINF	Macroeconomic stability measured by inflation rate	WDI
lnRES	Natural resources measured by the host country’s total rent of natural resources (% of GDP)	WDI
lnFAC	Infrastructure measured by average of fixed telephone users (per 100 people), fixed broadband users (per 100 people), and mobile cellular users (per 100 people)	WDI
lnTRA	Trade openness measured by import and export trade volume/GDP	WDI
lnFIN	Financial development measured by private sector domestic credit share/GDP	IMF

Table 2 provides the different statistics of all variables which describe our sample. We can conclude that the values of the variables are relatively stable, especially the standard deviations are relatively close and are within the controllable range, demonstrating that there are no outliers. Therefore, it is reasonable for us to select these variables for regression.

**Table 2:** Descriptive statistics of the variables.

Variables	Number of Samples	Average	Standard Deviation	Minimize	Maximize
lnOFDI	555	10.347	2.414	2.773	15.476
CC	555	-0.34	0.893	-1.681	2.248
GE	555	-0.1	0.908	-2.279	2.437
PS	555	-0.533	1.073	-3.006	1.616
RQ	555	-0.198	0.909	-2.268	2.261
RL	555	-0.273	0.879	-1.9	1.879
VA	555	-0.742	0.772	-2.259	1.11
WGI	555	-0.364	0.784	-2	1.63
lnGDP	555	25.28	1.712	21.561	29.467
lnDIS	555	12.643	0.529	10.873	13.641
lnLAB	555	4.099	0.211	3.606	4.476
lnFDI	555	0.791	1.228	-4.837	3.782
lnINF	555	1.488	1.203	-5.375	4.09
lnRES	555	0.886	2.783	-8.693	4.219
lnFAC	555	3.432	0.842	-0.842	4.478
lnTRA	555	4.158	0.537	2.995	5.839
lnFIN	555	3.637	0.957	0.442	5.159

## 4. Results and discussion

### 4.1. Preliminary inspection

In the first step, we employed the Pearson correlation coefficient matrix and the Variance Inflation Factor (VIF) to test for multicollinearity across all variables (S2 and S3 Appendices). The results of both tests showed no multicollinearity among all explanatory variables. In the second step, we proceed with the Hausman test and found that the fixed effects model should be used to estimate Eq (15).

The benchmark regression results are shown in Table 3. Columns (1)-(6) respectively demonstrate the impact of six sub-variables of governance quality on the OFDI of Chinese firms, including corruption control, government effectiveness, political stability, supervision quality, rule of law, voice and accountability in the host country. The results convey that corruption control, supervision quality, voice and accountability have a positive and statistically significant effect on OFDI, implying that increasing these three factors is not beneficial to attracting the inflow of OFDI. The coefficient of the overall governance quality shown in Column (7) is negative and statistically significant at the 1% level of significance, which confirms that the governance capacity of the host country has a significant inhibitory effect on the location selection of OFDI of Chinese enterprises. The result is consistent with Hypothesis 1b, but violates Hypothesis 1a. There may be two reasons: first, areas with a poor governance environment are often accompanied by corruption and rent-seeking behavior. Multinationals can take advantage of rent-seeking opportunities to facilitate the efficiency of administrative approvals and stimulate investment; second, during the sample period, Chinese firms have prepared countermeasures to invest in a harsh governance environment.

**Table 3:** Results of estimation by fixed effects model.

Variables	(1)	(2)	(3)	(4)	(5)	(6)	(7)
CC	-0.919***						
	(0.259)						
GE		-0.361					
		(0.264)					
PS			-0.0858				
			(0.152)				
RQ				-0.466*			
				(0.265)			
RL					-0.230		
					(0.319)		
VA						-0.969***	
						(0.252)	
WGI							-0.985***
							(0.356)
lnGDP	2.440***	2.433***	2.384***	2.428***	2.393***	2.243***	2.517***
	(0.205)	(0.215)	(0.216)	(0.210)	(0.216)	(0.205)	(0.214)
lnDIS	-0.634***	-0.602***	-0.568***	-0.631***	-0.578***	-0.597***	-0.687***
	(0.170)	(0.175)	(0.175)	(0.177)	(0.176)	(0.169)	(0.177)
lnLAB	6.562***	6.949***	6.904***	6.865***	7.019***	6.690***	6.444***
	(1.447)	(1.458)	(1.486)	(1.458)	(1.459)	(1.441)	(1.465)
lnFDI	-0.0706	-0.0824	-0.0764	-0.0811	-0.0812	-0.0638	-0.0614
	(0.0572)	(0.0577)	(0.0591)	(0.0577)	(0.0579)	(0.0572)	(0.0579)
lnINF	-0.0693	-0.0655	-0.0552	-0.0591	-0.0584	-0.0570	-0.0655
	(0.0461)	(0.0470)	(0.0465)	(0.0464)	(0.0466)	(0.0458)	(0.0463)
lnRES	-0.0701	-0.0713	-0.0900	-0.0618	-0.0891	-0.0955	-0.0523
	(0.0990)	(0.101)	(0.100)	(0.101)	(0.100)	(0.0985)	(0.100)
lnFAC	0.668***	0.626***	0.624***	0.666***	0.631***	0.769***	0.652***
	(0.117)	(0.117)	(0.119)	(0.119)	(0.118)	(0.121)	(0.117)
lnTRA	-0.0556	-0.209	-0.288	-0.135	-0.251	-0.142	-0.0705
	(0.280)	(0.283)	(0.276)	(0.290)	(0.283)	(0.274)	(0.285)
Constant	-72.38***	-73.14***	-71.80***	-72.81***	-72.57***	-68.78***	-73.12***
	(6.841)	(6.987)	(6.922)	(6.929)	(7.018)	(6.867)	(6.889)
Obs	555	555	555	555	555	555	555
R2	0.672	0.665	0.664	0.666	0.664	0.673	0.669

Note

*, ** and *** indicate the significance level at 10%, 5% and 1% respectively; Cluster-robust standard errors in parentheses. All these symbols are the same for the following tables.

**Fig 1:**
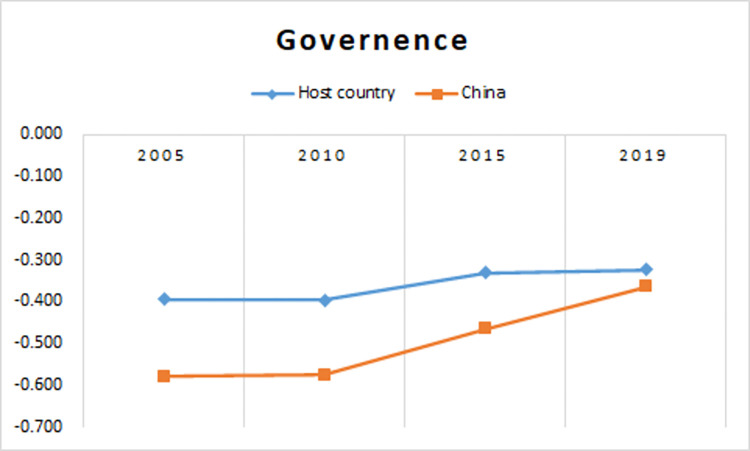
Governance line chart of Asian countries and China from 2005 to 2019.

In addition, we average the governance level of Asian countries selected in this paper and then draw a governance line chart of Asian countries and China from 2005 to 2019 (Fig 1). It can be found that although China’s governance is lower than that of Asia in the sample period, the gap between the two lines is gradually narrowing over time, and will disappear, especially in 2019. By comparing the governance of the two, we seem to be able to make a more reasonable explanation for the results of this article. Chinese firms have experienced a poor governance environment at home, so they have advantages in investing and surviving in countries with a low level of governance [75]. We need to realize that this paper seems to get an interesting result. Nevertheless, in the future, once China’s governance level exceeds that of Asia, we need to re-examine the theme discussed today.

For the control variables, the coefficient of the market size of the host country is considered positive, which means that Chinese companies make direct investments in Asia to seek a broader market, achieve economies of scale, and improve resource efficiency. Regarding bilateral distance cost, it negatively impacts the OFDI, which is consistent with the previous research [6, 76]. The coefficient of labor force size is positive and statistically significant at the 1% level of significance, proving that the comparative advantages of “great scale and low cost” in the labor force of Asian countries encourage OFDI. Regarding infrastructure, it has a positive impact on OFDI, implying that infrastructure is essential for OFDI. Although the coefficient of regional macroeconomic stability measured by the inflation rate is negative, it does not pass the 10% significance level, identifying that the macroeconomic stability of the Asian region is not strongly related to OFDI. Moreover, China’s direct investment in Asia did not concentrate on the host country’s opening-up level and resource endowments. This result echoes the research of Kamal et al. [5]. They found that the institutional quality in host countries affects the direction of China’s OFDI, but only in countries that are not rich in fuel resources. In contrast, the institutions of countries with rich fuel resources have little effect on the flow of Chinese OFDI.

### 4.2. Robustness test

#### 4.2.1. Replace the core explanatory variable

This paper refers to Habib and Zurawicki [77], using the principal component analysis method to measure the governance index, and the Eq (15) is performed again. Then KMO and SMC tests are also carried out to verify the suitability of principal component analysis, and the results reflect that it can play a better data reduction effect. The results are revealed in column (1) of Table 4. Compared with column (7) of Table 3, it is found that the coefficient of governance has not changed in sign and significance except for in size. In other words, even if the core explanatory variable measurement method is replaced, the host country’s governance still has a robust inhibitory effect on Chinese companies’ investment in Asia.

**Table 4:** Robustness test estimation results.

Variables	Full sample				Staging sample	
	Replace core explanatory variables	FGLS	Lag by one stage	2SLS	FE	2SLS
	(1)	(2)	(3)	(4)	(5)	(6)
WGI	-0.381***	-1.624***		-1.347***	-1.384**	-0.967***
	(0.136)	(0.146)		(0.153)	(0.667)	(0.259)
WGI_lag			-1.071***			
			(0.361)			
Control	Yes	Yes	Yes	Yes	Yes	Yes
Obs	555	555	518	518	185	148
R2	0.669	0.470	0.636	0.407	0.726	0.447

#### 4.2.2. Heteroskedasticity and serial correlation test

We employ the White test and Wooldridge test, respectively, to explain whether there are heteroscedasticity and serial correlation problems in variables. The results reject the null hypothesis of homoscedasticity and no autocorrelation. Therefore, this paper adopts feasible generalized least squares (FGLS) to estimate Eq (15), and the results are shown in column (2) of Table 4. Governance is significantly negatively correlated with Chinese firms’ OFDI in Asia, so the conclusions of this article are considered robust.

#### 4.2.3. Consider the lagging period of governance

Considering the influence of governance on OFDI of Chinese enterprises may have a certain lag, with reference to the method of Sun et al. [78], this inquiry makes a regression analysis again after the governance index lags behind one stage to verify the robustness of the benchmark regression. The results are shown in column (3) of Table 4. We can find that the lagged values still have a conspicuous negative effect on Chinese enterprises’ OFDI. Interestingly, when the governance lags for two and three periods, the coefficients are -1.222 and -1.027, respectively, which shows that the inhibitory effect of the lagging two periods is relatively stronger, confirming that the benchmark estimation results are robust under the influence of time lag.

#### 4.2.4. Endogeneity test

Endogeneity constitutes a critical problem for research as it undermines key conditions for claiming causality [79]. Considering governance may be endogenous, we referred to Hall and Jones [80], and adopted the distance index from countries to the equator and the lag of governance as instrumental variables. They argued that the distance to the equator reflects how deeply countries are influenced by the West, which can reflect changes in different institutions. Dunning proposed a classification for foreign direct investment (FDI) according to motivation, namely: (i) resource-seeking, (ii) market-seeking, (iii) efficiency-seeking, and (iv) strategic asset-seeking [81]. Therefore, we judged that the distance index from the host country to the equator would not directly affect China’s OFDI, which conforms to the principle of the externality of instrumental variables. Then we use the two-stage least squares method (2SLS) to deal with the endogeneity problem, and the estimation results are reported in column (4) of Table 4. Compared with the benchmark regression, the coefficient of governance rose from 0.985 to 1.347 in absolute value, which is consistent with the idea that the existence of endogenous problems seriously underestimated the inhibitory effect of governance on OFDI. In a word, the estimation results of this paper are proved to be robust again.

#### 4.2.5. Sample staging test

Governance has long-term implications. Therefore, we refer to the research method of Sheng and Jing [82], and process the samples in stages to investigate the impact of short-term economic cycle fluctuations on the results of this paper. Using FE and 2SLS, columns (5, 6) of Table 4 report the results with distance from the equator and lag values as instrumental variables, respectively. It can be seen that the coefficient of governance is still positive, and has passed the 5% significance level, which fully demonstrates that the benchmark results have strong robustness.

### 4.3. Mechanism test of financial development

Given the intermediary effect model proposed by Baron and Kenny [83], we construct a recursive model to explore the mechanism of host country governance affecting China’s foreign direct investment. If there is a vertical transmission process from WGI to financial development and then to OFDI, then the specific process is as follows:

1. It is necessary to verify that WGI can significantly affect OFDI, as demonstrated in the above benchmark regression and robustness analysis.
2. Try to prove that WGI influences financial development.
3. To confirm that WGI affects financial development and then influences OFDI.

Referring to the research methods of Guan and Xing [84], the model is as follows:

$${lnOFDI}_{it}=\gamma_{0}+\gamma_{1}{WGI}_{it}+\sum_{j}^{T}\gamma_{j=2}{control}_{it}^{j}+\varepsilon_{it}$$ (16)

$${lnFIN}_{it}=\alpha_{0}+\alpha_{1}{WGI}_{it}+\sum_{j=2}^{T}\alpha_{j}{control}_{it}^{j}+\varepsilon_{it}$$ (17)

$${lnOFDI}_{it}=\beta_{0}+\beta_{1}{WGI}_{it}+\beta_{2}{lnFIN}_{it}+\sum_{j=3}^{T}\beta_{j}{control}_{it}^{j}+\varepsilon_{it}$$ (18)

where lnFIN represents financial development approximated by private sector domestic credit as a share percentage of GDP [85]. Columns (1, 4) of Table 5 present the results of Eq (16), which verifies the role of WGI on China’s direct investment in Asia. Eq (17) examines the impact of governance on financial development, and the results are shown in columns (2, 5) of Table 5. Eq (18) detects the effect of financial development on China’s direct investment in Asia, and the results are reported in columns (3, 6) of Table 5.

**Table 5:** Mechanism test estimation results.

	FGLS			2SLS		
Variables	lnOFDI	lnFIN	lnOFDI	lnOFDI	lnFIN	lnOFDI
	(1)	(2)	(3)	(4)	(5)	(6)
WGI	-1.624***	0.355***	-1.408***	-1.348***	0.432***	-0.903***
	(0.146)	(0.0354)	(0.153)	(0.174)	(0.0529)	(0.175)
lnFIN			-0.678***			-1.030***
			(0.142)			(0.137)
Control	Yes	Yes	Yes	Yes	Yes	Yes
Obs	555	555	555	518	518	518
R2	0.470	0.772	0.495	0.407	0.672	0.467

Indeed, it can be seen from columns (2, 5) that governance is significant and positively correlated with financial development. It suggests that countries with good institutional environments have more advantages in their financial markets regarding information processing, capital allocation, and risk management. Financial development is negatively and considerably correlated with OFDI (columns (3, 6)), and the absolute value of the governance coefficient has declined compared with columns (1, 4), which indicates that financial development is an important channel for the governance of the host country to influence China’s firms to invest in Asia. Thus, Hypothesis 2b has been confirmed, but it deviates from Hypothesis 2a according to the coefficient signs of the variable.

### 4.4 Further discussion

#### 4.4.1. Quantile test

This paper employs a quantile regression approach to estimate 10%, 50%, and 90% of the quantile, respectively, to detect the effect of governance on different levels of direct investment in China. The results are reported in columns (1–3) of Table 6. The coefficients of governance are all negative, which also justifies the robustness of the benchmark estimation results. The absolute value of the coefficient increases as the OFDI booms. In other words, the impact of governance on Chinese FDI in Asia increases with the size of OFDI. However, when OFDI increases to a certain extent, the role of governance on OFDI will be decreased. Two possible reasons can be explained: on the one hand, large-scale OFDI occurs in areas with higher economic development, and multinationals are relatively weak in response to governance to seek a broad market. On the other hand, the huge OFDI scale facilitates multinationals to form economies of scale, continuously optimize the allocation efficiency of corporate funds, provide more jobs for the host country, and better integrate into the host country’s institutional environment.

**Table 6:** Estimated results for further discussion.

	Quantile test			The BRI	Placebo test
Variables	10%	50%	90%		
	(1)	(2)	(3)	(4)	(5)
WGI	-0.480*	-0.750***	-0.287**	-1.265***	-0.902***
	(0.288)	(0.211)	(0.116)	(0.205)	(0.235)
BR				1.754***	2.366***
				(0.188)	(0.192)
WGI×BR1				0.511***	
				(0.196)	
WGI×BR2					0.187
					(0.208)
Control	Yes	Yes	Yes	Yes	Yes
Obs	555	555	555	518	518
R2	0.490	0.395	0.419	0.493	0.547

#### 4.4.2. The impact of the “Belt and Road” initiative

In order to characterize whether the introduction of the BRI in 2013 has changed the influence of governance on China’s OFDI in Asia, we draw into the intersection of governance and the BRI dummy variables, and construct the following equation:

$${lnOFDI}_{it}=\tau_{0}+\tau_{1}{WGI}_{it}+\tau_{2}{road}_{it}+\rho WGI\times {road}_{it}+\sum_{j}^{T}\tau_{j=3}{control}_{it}^{j}+\varepsilon_{it}$$ (19)

when *t*≥2013, road = 1, otherwise, road = 0. The index of the virtual variable is found to be significant and positively correlated with China’s OFDI in column (4) of Table 6, implying that the proposed initiative can promote the direct investment of Chinese enterprises in Asia. Yu et al. pointed out that the BRI had improved China’s OFDI for the developing countries that supported it [86]. The interaction between governance and the BRI is also positive, indicating that the BRI plays a modulator effect on the role and weakens the inhibitory effect of governance on OFDI. Shao also noted that the BRI could alleviate the negative impact of the host country’s political risks on Chinese enterprises OFDI [87]. The possible reason is that the BRI could provide institutional protection for companies to invest in Asian countries with low governance capabilities.

#### 4.4.3. Placebo test

We use the placebo test to verify the robustness of the BRI effectiveness. Specifically, we set the virtual variable as the critical point in 2010, reconstructing the interaction with governance, and the results are presented in column (5) of Table 6. The interaction term coefficient is greatly reduced compared to column (4). Although it is positive, it does not pass the significance level test. For the sake of prudence, we also selected 2008 and 2011 to do the same test, and the results still support the above conclusions.

### 4.5. Heterogeneity analysis of geographical characteristics and economic development

#### 4.5.1. Classification of China’s neighbors and non-neighbors

According to the expanded gravity model, the distance cost between the two countries is an extremely important factor affecting multinationals’ OFDI. Scholars have conducted many studies on the relationship between geographic distance and international direct investment [88, 89]. We divide Asian countries into neighbors and non-neighbors according to whether they are adjacent to China’s border or whether they have a common border. Columns (1, 2) of Table 7 report the results. Governance is significant and negatively correlated with OFDI in both groups. The absolute value of the coefficient of neighbors is greater than that of non- neighbors, which proves that the governance of China’s neighbors has a more obvious impact on China’s foreign direct investment in Asia. The possible explanation is that there is a spatial spillover effect between the governance of neighbors, and the OFDI of enterprises will also be largely affected by the domestic institutions [90].

**Table 7:** Heterogeneity estimation results.

Variables	neighbors	non-neighbors	Inland	Coastal	Low-income	High-income
	(1)	(2)	(3)	(4)	(5)	(6)
WGI	-1.997***	-1.351***	0.430	-1.299***	-2.696***	-0.411
	(0.341)	(0.197)	(0.537)	(0.193)	(0.358)	(0.259)
Control	Yes	Yes	Yes	Yes	Yes	Yes
Obs	154	364	126	392	154	238
R2	0.661	0.510	0.684	0.468	0.690	0.679

#### 4.5.2. Division of inland and coastal countries

Except for Japan and South Korea, the Asian sample countries selected in this paper are all located along the BRI, including the land-based and the maritime silk road economic belt. There are significant differences between the two types of countries regarding macroeconomics and national institutions [91]. This inquiry divides the selected samples into landlocked and coastal countries. Columns (3, 4) of Table 7 report the results. We find a negative and significant relationship between our measure of governance and China’s OFDI in coastal countries. In contrast, the coefficient of inland countries is positive. However, it does not pass the 10% significance, demonstrating that Chinese corporations do not pay much attention to the governance capacity of the host country when investing in landlocked countries.

#### 4.5.3. Division of economic development level of the host country

We draw on the methods of Wang et al. [92], and divide the samples into high- and low-income countries according to per capita national income. Columns (5, 6) of Table 7 show the estimated results. The results show that two types of estimation results are negatively correlated, but they are only significant in low-income countries. This phenomenon may be because low-income countries will hide some investment opportunities, such as providing rent-seeking opportunities for investors to improve investment efficiency; formulating preferential policies for multinationals to attract capital inflows. Compared with low-income, high-income countries have a better business and institutional environment. Chinese firms care more about the host country’s market when investing in such countries.

## 5. Conclusions

This paper systematically examines the relationship between governance, financial development, and OFDI by constructing a model considering multinationals and governance heterogeneity. On this basis, using panel data of China’s direct investment in Asian countries from 2005 to 2019 to conduct empirical tests. The main conclusions of this paper are as follows:

1. In the benchmark regression, the governance of the host country is negatively correlated with China’s direct investment in Asia. Besides, after replacing the core explanatory variables, considering heteroscedasticity and sequence-related issues, and employing the distance from countries to the equator as an instrumental variable to overcome the endogenous problem and avoid the interference of economic fluctuations, our findings convey that the conclusions are still robust. In addition, through the analysis of the intermediary effect model, financial development is an important channel for the governance of the host country to affect China’s OFDI.
2. With the changes in the scale of China’s OFDI, the governance of the host country also shows heterogeneity. As the scale of OFDI expands, the role of governance takes an inverted “U” shape. Furthermore, we examine the effect of exogenous factors of the BRI, which has weakened the negative influence of governance on China’s OFDI.
3. In the heterogeneity test, governance has shown more remarkable restraint on China’s OFDI in neighboring, coastal, and low-income countries.

We put forward relevant policy recommendations based on the above research conclusions. Strengthening the interconnection with Asian countries will help multinationals increase their familiarity with their rules and regulations. Even if the investment preference of Chinese firms in Asia flows to countries with low governance levels in the short term, a better governance environment should be taken as the condition for the optimal location choice of multinationals’ OFDI in the long run. The Chinese government should improve its information service system and establish an efficient information collection and dissemination platform. Timely and accurately release of the governance information of the host country, to provide information reference for investment enterprises to make foreign direct investment decisions. By obtaining sufficient information, multinational corporations can reduce the investment risk caused by information asymmetry.

Moreover, through the research of this paper, efforts should be made by the Chinese government to stimulate the BRI construction, improve the terms of the investment protection agreement, and create a safe and transparent market environment for global businesses to invest in developing countries with poor governance. Last on, before investing abroad, enterprises should not only rely on the macro risk assessment index made by the government or institutions, but also internalize the index from the micro-level. Once the risk index exceeds expectations, they should take timely countermeasures to avoid more significant benefit losses.

## Supporting information

S1 Appendix China’s direct investment stock in Asian countries from 2005 to 2019. (DOCX)

S2 Appendix Pearson correlation coefficient matrix. (DOCX)

S3 Appendix Results of the VIF test. (DOCX)

S1 Data (XLSX)

S1 File (DO)
