# Supplementary material for: Governance, financial development and China’s outward foreign direct investment
Source: PLoS One. 2022 Jun 30;17(6):e0270581. doi: 10.1371/journal.pone.0270581 (PMC9246154; doi:10.1371/journal.pone.0270581)
Supplement: S1 Appendix — (DOCX) [file pone.0270581.s001.docx]

**S1 Appendix. China's direct investment stock in Asian countries from 2005 to 2019**

**(Unit: USD 100 million ,%)**

| **Country** | **2005** | | **2010** | | **2015** | | **2019** | |
| --- | --- | --- | --- | --- | --- | --- | --- | --- |
|  | **OFDI** | **%** | **OFDI** | **%** | **OFDI** | **%** | **OFDI** | **%** |
| Uzbekistan | 1198 | 0.33 | 8300 | 0.31 | 88204 | 0.84 | 324621 | 1.89 |
| Yemen | 7777 | 2.15 | 18466 | 0.69 | 45330 | 0.43 | 54419 | 0.32 |
| Israel | 632 | 0.17 | 2187 | 0.08 | 31718 | 0.30 | 377502 | 2.19 |
| Iraq | 43487 | 12.03 | 48345 | 1.82 | 38812 | 0.37 | 137752 | 0.80 |
| Iran | 5608 | 1.55 | 71516 | 2.69 | 294919 | 2.82 | 305562 | 1.78 |
| Qatar | 270 | 0.07 | 7705 | 0.29 | 44993 | 0.43 | 45892 | 0.27 |
| India | 1462 | 0.40 | 47980 | 1.81 | 377047 | 3.61 | 361009 | 2.10 |
| Indonesia | 14093 | 3.90 | 115044 | 4.33 | 812514 | 7.78 | 1513255 | 8.79 |
| Kyrgyzstan | 4506 | 1.25 | 39432 | 1.48 | 107059 | 1.02 | 155003 | 0.90 |
| Kazakhstan | 24524 | 6.78 | 159054 | 5.98 | 509546 | 4.88 | 725413 | 4.22 |
| Turkmenistan | 20 | 0.01 | 65848 | 2.48 | 13304 | 0.13 | 22656 | 0.13 |
| Turkey | 423 | 0.12 | 40363 | 1.52 | 132884 | 1.27 | 186786 | 1.09 |
| Tajikistan | 2279 | 0.63 | 19163 | 0.72 | 90909 | 0.87 | 194608 | 1.13 |
| Korea | 88222 | 24.40 | 63725 | 2.40 | 369804 | 3.54 | 667340 | 3.88 |
| Bangladesh | 3296 | 0.91 | 6758 | 0.25 | 18843 | 0.18 | 124830 | 0.73 |
| Nepal | 299 | 0.08 | 1594 | 0.06 | 29193 | 0.28 | 53866 | 0.31 |
| Pakistan | 18881 | 5.22 | 182801 | 6.88 | 403593 | 3.86 | 479798 | 2.79 |
| Bahrain | 199 | 0.06 | 87 | 0.00 | 387 | 0.00 | 7074 | 0.04 |
| Brunei | 190 | 0.05 | 4566 | 0.17 | 7352 | 0.07 | 42696 | 0.25 |
| Sri Lanka | 1543 | 0.43 | 7274 | 0.27 | 77251 | 0.74 | 55147 | 0.32 |
| Singapore | 32548 | 9.00 | 606910 | 22.84 | 3198491 | 30.61 | 5263656 | 30.59 |
| Japan | 15070 | 4.17 | 110563 | 4.16 | 303820 | 2.91 | 409805 | 2.38 |
| Cambodia | 7684 | 2.13 | 112977 | 4.25 | 367586 | 3.52 | 646370 | 3.76 |
| Saudi Arabia | 5845 | 1.62 | 76056 | 2.86 | 243439 | 2.33 | 252773 | 1.47 |
| Thailand | 21918 | 6.06 | 108000 | 4.06 | 344012 | 3.29 | 718585 | 4.18 |
| Kuwait | 123 | 0.03 | 5087 | 0.19 | 54362 | 0.52 | 83451 | 0.49 |
| Jordan | 1747 | 0.48 | 1263 | 0.05 | 3255 | 0.03 | 31173 | 0.18 |
| Myanmar | 3227.84 | 0.89 | 194675 | 7.32 | 425873 | 4.08 | 413445 | 2.40 |
| Laos | 3287 | 0.91 | 84575 | 3.18 | 484171 | 4.63 | 824959 | 4.79 |
| Philippines | 1935 | 0.54 | 38734 | 1.46 | 71105 | 0.68 | 66409 | 0.39 |
| Mongolia | 13063 | 3.61 | 143552 | 5.40 | 376006 | 3.60 | 343054 | 1.99 |
| Vietnam | 2326.84 | 0.64 | 98660 | 3.71 | 337356 | 3.23 | 707371 | 4.11 |
| Afghanistan | 45 | 0.01 | 16859 | 0.63 | 41993 | 0.40 | 41894 | 0.24 |
| The United Arab Emirates | 14453 | 4.00 | 76429 | 2.88 | 460284 | 4.41 | 763567 | 4.44 |
| Oman | 653 | 0.18 | 2111 | 0.08 | 20077 | 0.19 | 11634 | 0.07 |
| Malaysia | 18683 | 5.17 | 70880 | 2.67 | 223137 | 2.14 | 792369 | 4.61 |
| Lebanon | 17 | 0.00 | 201 | 0.01 | 378 | 0.00 | 222 | 0.00 |
