# Supplementary material for: Governance, financial development and China’s outward foreign direct investment
Source: PLoS One. 2022 Jun 30;17(6):e0270581. doi: 10.1371/journal.pone.0270581 (PMC9246154; doi:10.1371/journal.pone.0270581)
Supplement: S2 Appendix — (DOCX) [file pone.0270581.s002.docx]

**S2 Appendix. Pearson correlation coefficient matrix**

| **Variable** | **lnGDP** | **lnDIS** | **lnLAB** | **lnFDI** | **lnINF** | **lnRES** | **lnFAC** | **lnTRA** | **WGI** | **lnFIN1** |
| --- | --- | --- | --- | --- | --- | --- | --- | --- | --- | --- |
| lnGDP | 1 |  |  |  |  |  |  |  |  |  |
| lnDIS | -0.00770 | 1 |  |  |  |  |  |  |  |  |
| lnLAB | -0.00180 | -0.302 | 1 |  |  |  |  |  |  |  |
| lnFDI | -0.303 | 0.0466 | 0.0801 | 1 |  |  |  |  |  |  |
| lnINF | -0.267 | 0.180 | -0.129 | 0.0566 | 1 |  |  |  |  |  |
| lnRES | -0.216 | 0.183 | 0.114 | -0.0668 | 0.331 | 1 |  |  |  |  |
| lnFAC | 0.410 | 0.0955 | 0.131 | 0.0462 | -0.375 | -0.108 | 1 |  |  |  |
| lnTRA | -0.199 | 0.0383 | 0.346 | 0.510 | -0.0667 | 0.0264 | 0.262 | 1 |  |  |
| WGI | 0.473 | -0.127 | 0.372 | 0.0727 | -0.386 | -0.361 | 0.583 | 0.381 | 1 |  |
| lnFIN | 0.501 | -0.162 | 0.334 | 0.0422 | -0.364 | -0.369 | 0.594 | 0.305 | 0.760 | 1 |
