# Supplementary material for: Governance, financial development and China’s outward foreign direct investment
Source: PLoS One. 2022 Jun 30;17(6):e0270581. doi: 10.1371/journal.pone.0270581 (PMC9246154; doi:10.1371/journal.pone.0270581)
Supplement: S3 Appendix — (DOCX) [file pone.0270581.s003.docx]

**S3 Appendix. Results of the VIF test**

| **Variable** | **VIF** | **1/VIF** |
| --- | --- | --- |
| WGI | 3.28 | 0.305225 |
| lnFIN | 3.07 | 0.325759 |
| lnTRA | 2.06 | 0.485926 |
| lnFAC | 2.01 | 0.497051 |
| lnGDP | 1.98 | 0.504175 |
| lnLAB | 1.60 | 0.625466 |
| lnRES | 1.59 | 0.628734 |
| lnFDI | 1.49 | 0.669490 |
| lnINF | 1.37 | 0.370346 |
| lnDIS | 1.27 | 0.789197 |
| Mean VIF | 1.97 | 0.507614 |
